# Flucrypyrim, a novel uterine relaxant, has antinociceptive and anti-inflammatory effects *in vivo*

**DOI:** 10.1038/srep42040

**Published:** 2017-02-21

**Authors:** Zhongtang Li, Limei Wang, Yue Cong, Lin Guo, Xiaohui Lin, Zuyin Yu, Xingan Wu, Junxing Dong, Rifang Yang, Yuwen Cong

**Affiliations:** 1 Department of Pathophysiology, Beijing Institute of Radiation Medicine, Beijing, China; 2 Department of Medicinal Chemistry, Beijing Institute of Pharmacology and Toxicology, Beijing, China; 3 Department of Microbiology, Fourth Military Medical University, 17 Changle Xi Road, Xi’an, Shaanxi Province, 710032, PR China; 4 Department of Pharmaceutical Sciences, Beijing Institute of Radiation Medicine, Beijing, China

## Abstract

Consequences of primary dsysmenorrhea (PD) can be severe. Increased prostaglandin production leads to uterine contraction and insufficient blood flow to the endometrium causing ischemia and pain symptoms. Protein tyrosine kinase/phosphatase activities contribute to the modulation of uterine contraction. In our previous study, we found the synthetic β-methoxyacrylates compound Fluacrypyrim (FAPM), significantly increased protein tyrosine phosphatases (PTPs) activity, resulting in dephosphorylation of tyrosine kinases. In the present study, we found that FAPM near completely inhibited prostaglandin F2α (PGF_2α_)-, oxytocin-, acetylcholine-, and high K^+^-induced uterine contractions in rats *in vitro,* and decreased rat myometrial myosin light chain (MLC_20_) phosphorylation induced by PGF_2α_. A structure–activity relationship assay indicated that the β-methoxyacrylates structure of FAPM is crucial for the inhibition of PGF_2α_-induced uterine contractions. FAPM caused a concentration-dependent parallel rightward shift of the concentration–response curve induced by oxytocin, dose-dependently reduced the number of abdominal constrictions and increased the latency time in PGF_2α_- and acetic acid-induced writhing test in mice *in vivo*. Furthermore, FAPM considerably inhibited the development of Carr-induced rat paw edemas and thexylene-induced mouse ear edemas. Taken together, our results indicate that FAPM exerts antinociceptive and anti-inflammatory effects *in vivo* with considerable potential as a novel uterine relaxant.

Primary dysmenorrhea (PD), or painful menses, is considered one of the most common gynecological complaints among adolescent and young adult women^1^. It is characterized by menstrual cramps and lower abdominal pain and may be associated with nausea, vomiting, diarrhea, headache, dizziness, and/or back pain. The prevalence of PD is highest among adolescent women, affecting from 20 to 90 of this age group^2^. Moreover, ~15% percent of adolescent girls suffer from such severe dysmenorrhea, that this condition represents the leading cause of school and work absenteeism^3^. Notably, the cause of PD still remains unclear^4^.

It is believed that prostaglandin (PG) release plays an important role in the pathogenesis of dysmenorrhea. PGF_2α_, a cyclooxygenase (COX) metabolite of arachidonic acid, have also been shown to have important implications to this disease^5^. Previous studies have reported that women with PD have higher endogenic PGF_2α_ levels as compared to their asymptomatic counterparts^6^. PGF_2α_ stimulates vasoconstriction and uterine contractions, leading to ischemia and the pain symptoms associated with PD^7^. Nonsteroidal anti-inflammatory drugs (NSAID) inhibit the synthesis of PGs, and are considered first-line therapy for this condition^8^. While NSAIDs successfully treats PD in the majority of cases, patients continue to suffer from adverse long-term effects involving disorders of the liver, digestive systems and kidney^9^. Oral contraceptive pills (OCPs) represent another treatment modality, but is less frequently used due to the implications for pregnancy^10^. Despite the successes obtained via pharmacologic therapies, there still exists a 20% to 25% treatment failure rate^11^.

Recently, studies have shown that protein tyrosine kinase/phosphatase activities control both phosphorylation and activation of signaling proteins including: phospholipase C-γ, Ca^2+^-dependent tyrosine kinase Pyk2, c-Src and Lck kinases. Together these signaling pathways modulate uterine contractions induced by contractile agonists such as PGF_2α_^12^,^13^,^14^. More specifically, the tyrosine kinase inhibitor genistein, by example, released uterine contractions elicited by pervanadate, a protein tyrosine phosphatase inhibitor, which can potentiate contractile receptor-mediated contraction^15^,^16^. Similarly, Imatinib mesylate suppresses the contractile activity of human uterus by inhibiting the tyrosine kinase activity of c-kit, PDGFR, and abl^17^,^18^. Tyrosine phosphorylation results from the dynamic equilibrium between phosphorylation and dephosphorylation reactions, the latter of which is regulated through the activity of protein tyrosine phosphatases (PTPs)^19^. In our previous work, we found fluacrypyrim (FAPM), identified as a potent STAT3 activation inhibitor, significantly increases PTPs activity in a dose-dependent manner. Furthermore, FAPM-induced suppression of STAT3 tyrosine phosphorylation can be reversed via sodium pervanadate, which highlights the importance of FAPM in regulating PTPs^20^. In the study described herein, we examined the direct effects of FAPM on uterine contraction in a rat model and evaluated the analgesic and anti-inflammatory effects *in vivo*. Moreover, we report a novel, inhibitory role of FAPM on rat uterine contractions induced by stimulators and evaluate the analgesic and anti-inflammatory activities of this agent *in vivo*.

## Results

### Effects of FAPM and its analogs on PGF_2α_-induced uterine contractions in the rats

**Figure 1:**
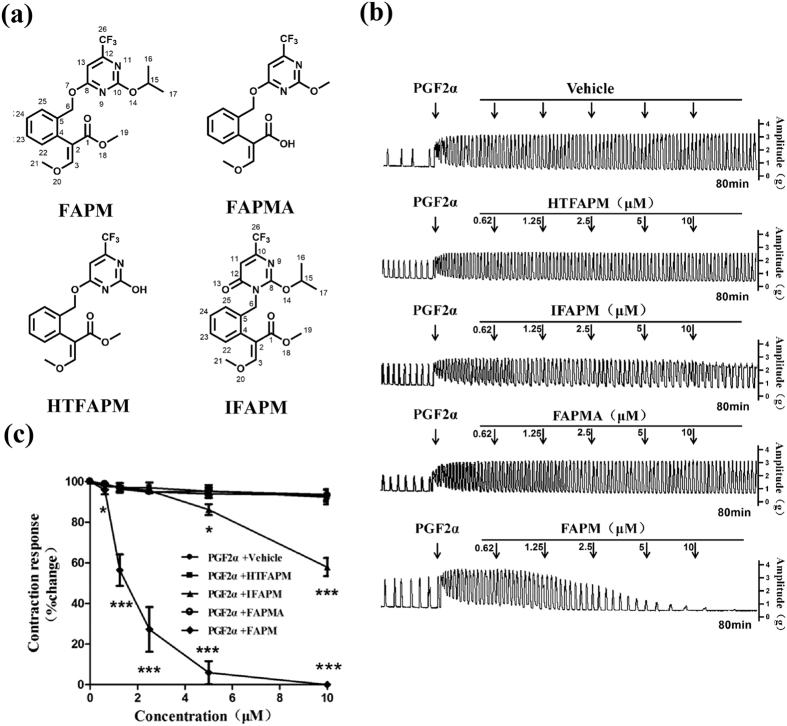
FAPM inhibits PGF_2α_-induced uterine contraction in rats. Rat uterine segments were treated with PGF_2α_ and exposure of rat uterine smooth muscles to vehicle (DMSO, 20%), FAPM or its analogs. (**a**) Structures of FAPM or its analogs. (**b**) Representative recordings of PGF_2α_ induced contractions treated with vehicle only, FAPM and its analogs. (**c**) Dose-effect curve of FAPM and its analogs on PGF_2α_-induced uterine contraction. The values represent the mean ± S.E.M. (n = 3 to 5); *P < 0.05; ***P < 0.001 *vs.* control (vehicle) group.

To investigate the *in vivo* inhibition of PGF_2α_-induced uterine contraction by FAPM, we first examined the effect of FAPM on PGF_2α_-induced uterine contraction *in vitro*. As shown in Fig. 1b, application of nanomolar concentration of PGF2α (450 nM final concentration in the bath solution) produced phasic contractions of constant amplitude and frequency. FAPM exerted a relaxant effect on PGF_2α_-induced uterine contraction in a concentration-dependent manner (0.625~10 μmol/L). A representative recording of the inhibition of both frequency and amplitude of contractions induced by FAPM is shown in Fig. 1c. The contractions expressed as percentage of the response to 450 nM PGF_2α_ were measured as the AUC at 10-min intervals to characterize the inhibitory activity of FAPM. On the basis of the dose–response curve (Fig. 1b), FAPM induced a complete (100%) reduction in contractility *vs.* 6.41 ± 2.68% for Vehicle (*P* < 0.001), with the average pD_2_ value of (−log10 of the concentration of FAPM achieving half maximal inhibition) 5.72.

To characterize the structure of FAPM, a structure–activity relationship assay was performed using FAPM analogs against PGF_2α_-induced uterine contraction (Fig. 1a). As showed in Fig. 1b, removing the isopropyl group at O14-site in FAPM (HTFAPM) or hydrolyzing the methyl acrylate of FAPM into acrylic acid (FAPMA) both leads to non-significant inhibition against PGF_2α_-induced uterine contraction. When we changed the connection mode of benzene and pyrimidine in FAPM, where the benzyl was moved to N9-position from O7-position (IFAPM), the inhibitory activity of IFAPM was significant (*P* < 0.001), but lower than that of FAPM. On the basis of the dose–response curve (Fig. 1c), the net maximum reduction in contractility for HTFAPM, FAPMA and IFAPM was 7.72 ± 3.52%, 6.84 ± 3.11% and 42.15 ± 4.49% respectively. The structure–activity relationship assay indicated that both methyl group (C19) at O18-position and isopropyl group at O14-site in FAPM are critical for the effect, while the bonding site of arylmethyl group to the pyrimidine is also critical.

### Effects of FAPM on oxytocin-induced uterine contractions in rats

**Figure 2:**
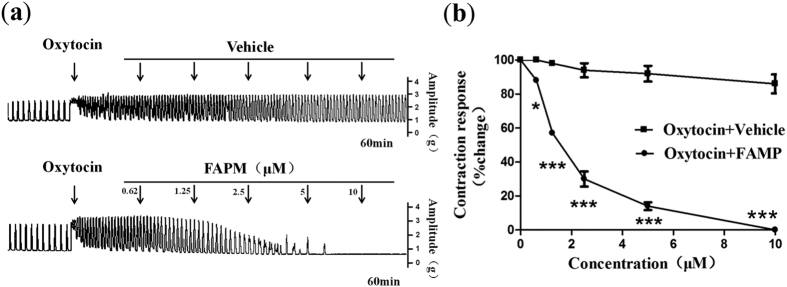
FAPM inhibits oxytocin-induced contraction in rats. Rat uterine segments were treated with oxytocin and exposure of rat uterine smooth muscles to vehicle (DMSO, 20%) or FAPM. (**a**) Representative recording of FAPM on contractions of rat uterus induced by oxytocin. (**b**) Dose-effect curve of FAPM on contractions of rat uterus induced by oxytocin. The values represent the mean ± S.E.M. (n = 5); *P < 0.05; ***P < 0.001 *vs.* control (vehicle) group.

Oxytocin is a nonapeptide hormone that stimulates uterine and mammary gland contractions via oxytocin receptor^21^. To determine whether FAPM exerted an inhibitory effect on oxytocin-induced contractions, different doses of FAPM were administered along with oxytocin (1 mU/ml). As showed in Fig. 2a, FAPM exerted an inhibitory effect on uterine contractile force (amplitude) and frequency. On the basis of the dose–response curve (Fig. 2b), the percentage of net maximum reduction in contractility for FAPM was 100% *vs.* 13.81 ± 2.68% for Vehicle (*P* < 0.001), with the average pD_2_ value of 5.79.

**Figure 3:**
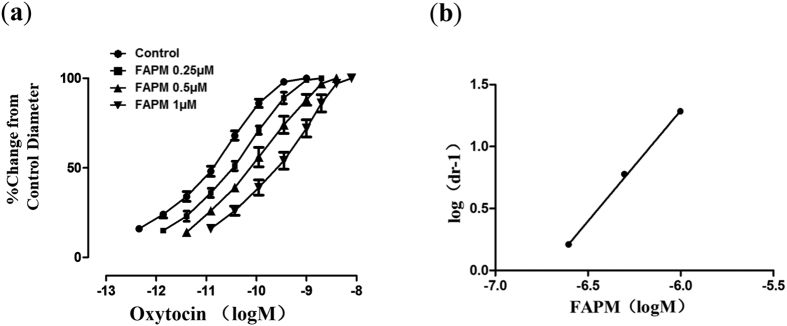
Antagonism of oxytocin-induced contraction by FAPM in isolated rat uterine. (**a**) Concentration-response curves to oxytocin in the absence or presence of increasing concentrations of FAPM. (**b**) The curve of the Schild plot with the slope values of 1.8 ± 0.34 and pA_2_ values of 6.72 ± 0.03. Each point represents the mean ± S.E.M. (n = 3).

To characterize the antagonism of oxytocin-induced contraction by FAPM, increasing doses of oxytocin were administered along with FAPM. As showed in Fig. 3a, the presence of FAPM caused a concentration-dependent parallel rightward shift without any reduction in maximal contractile response. The slope and pA_2_ values obtained from the Schild plot analysis were 1.78 ± 0.34 and 6.72 ± 0.03, respectively, indicating a competitive antagonism by FAPM against the oxytocin-induced contraction (Fig. 3b).

### *In vitro* analysis of the effects of FAPM on acetylcholine and KCl -induced uterine contractions

**Figure 4:**
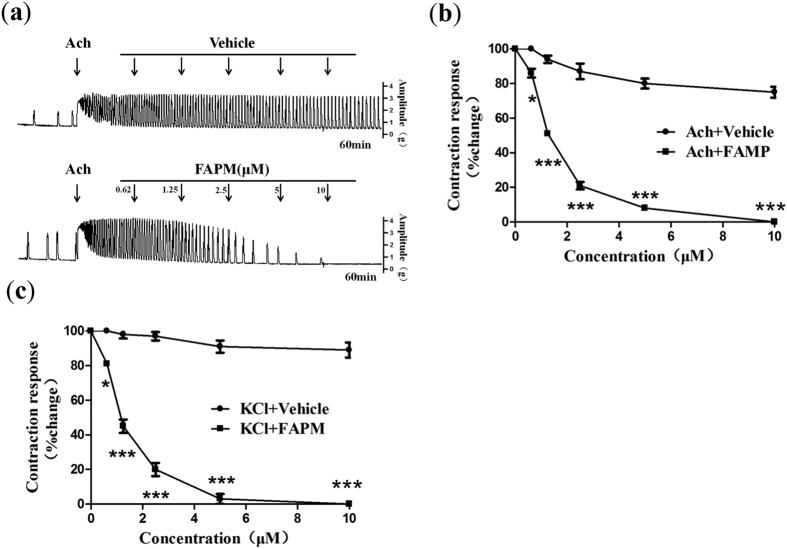
FAPM inhibits Ach, and KCl-induced contraction in rats. Rat uterine segments were treated with Acetylcholine (Ach) or KCl and exposure of rat uterine smooth muscles to vehicle (DMSO, 20%) or FAPM. (**a**) Representative recording of FAPM on contractions of rat uterus induced by Acetylcholine. (**b**) Dose-effect curve of FAPM on contractions of rat uterus induced by Acetylcholine. (**c**) Dose-effect curve of FAPM on contractions of rat uterus induced by KCl. The values represent the mean ± S.E.M. (n = 4 to 5); *P < 0.05; ***P < 0.001 *vs.* control (vehicle) group.

Acetylcholine (Ach) behaves as an excitatory neurotransmitter at neuromuscular junctions in uterine smooth muscle. As shown in Fig. 4a, application of 0.25 μM Ach produced phasic contractions of constant amplitude and frequency, and administration of FAPM along with Ach exerted an inhibitory effect on uterine contractile force and frequency. Based on the dose–response curve (Fig. 4b), the percentage of net maximum reduction in contractility for FAPM was 100% *vs.* 25.21 ± 3.15% for Vehicle (*P* < 0.001), with the average pD_2_ value of 5.86.

Previous reports have suggested that high-K^+^ depolarizing solution can induce uterine contractions. Therefore, we investigated the effects of FAPM on K^+^ depolarization-induced uterine contractions. The administration of different doses of FAPM along with KCl (16 mM) resulted in a dose-dependent inhibition of uterine contraction. On the basis of the dose–response curve (Fig. 4c), the percentage of net maximum reduction in contractility for FAPM was 100% *vs.* 10.58 ± 4.28% for Vehicle (*P* < 0.001), with the average pD_2_ value of 5.92.

### Effects of FAPM on PGF_2α_-induced MLC_20_ phosphorylation

**Figure 5:**
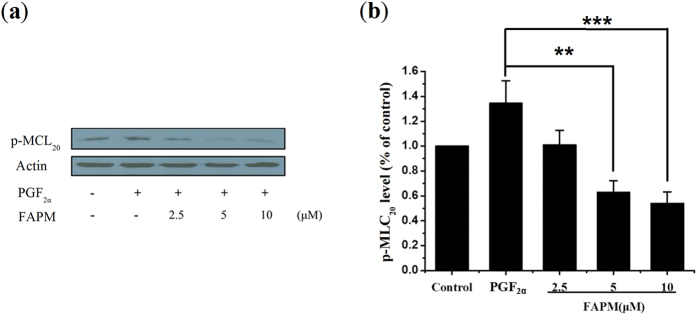
FAPM inhibits PGF_2α_-induced MLC_20_ phosphorylation. (**a**) Myometrial cells were treated with the indicated concentrations of FAPM, total cell lysates were prepared and examined for p-MLC_20_ protein levels by Western blot analysis using the respective antibodies. Actin was used as a protein loading control. (**b**) half-quantification of the western blot. Each column represents the mean ± S.E.M. (n = 3). **P < 0.01; ***P < 0.001 *vs.* PGF_2α_ group.

Phosphorylation of MLC_20_ primarily regulated by calcium- and calmodulin-dependent myosin light chain kinase (MLCK) is a key regulator of smooth muscle contraction^22^. To study whether FAPM inhibits MLC_20_ phosphorylation, rat myometrial cells was treated with FAPM (2.5, 5, and 10 μM) along with PGF_2α_ (10^−6^ m). As shown in Fig. 5, the application of PGF_2α_ for 5 min induced MLC_20_ phosphorylation, and FAPM treatment (5 and 10 μM) significantly reduced the PGF_2α_-induced MLC_20_ phosphorylation in a dose-dependent manner.

### Analgesic activity of FAPM on the acetic acid-induced writhing test

**Figure 6:**
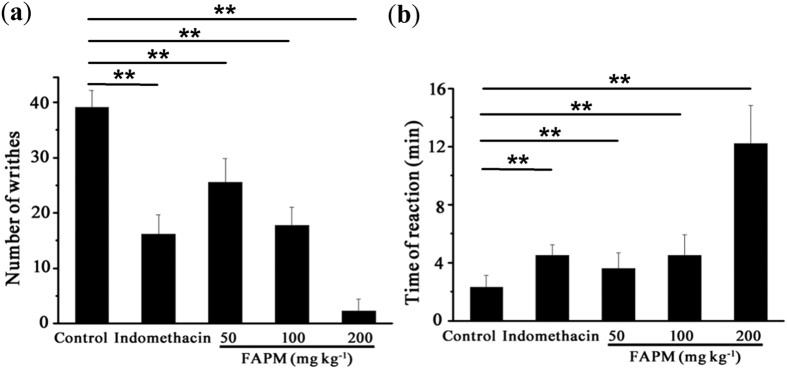
FAPM reduces acetic acid-induced writhing response. Mice were treated with indomethacin, FAPM or the same volume of vehicle (DMSO, 20%) 1 h before injection of 0.6% aqueous solution of acetic acid. (**a**) The number of writhes was counted for 30 min after acetic acid administration. (**b**) The time to onset of writhing was recorded after acetic acid administration. Data were presented as the mean ± S.E.M. (n = 10). **p < 0.01 *vs.* control (vehicle) group.

The writhing test was used to evaluate the analgesic activity of FAPM. Administration of FAPM (50, 100 and 200 mg/kg) 1 h before acid injection produced a significant and dose-dependent inhibition of acetic acid-induced abdominal constrictions in mice (Fig. 6a). The latency time was also found to be increased significantly in a dose dependent manner in the FAPM group (Fig. 6b). FAPM (100 mg/kg) demonstrated a significant inhibition of acetic acid-induced writhing response, similar to that of indomethacin (50 mg/kg), a standard NSAID used as positive control. The FAPM group (200 mg/kg) exhibited the maximum inhibiting effect of acetic acid-induced writhing and the longest latency time (12.2 min) - almost six fold long as that in the vehicle control group (2.2 min).

### Anti-inflammatory activities of FAPM in mouse and rat swelling models

**Figure 7:**
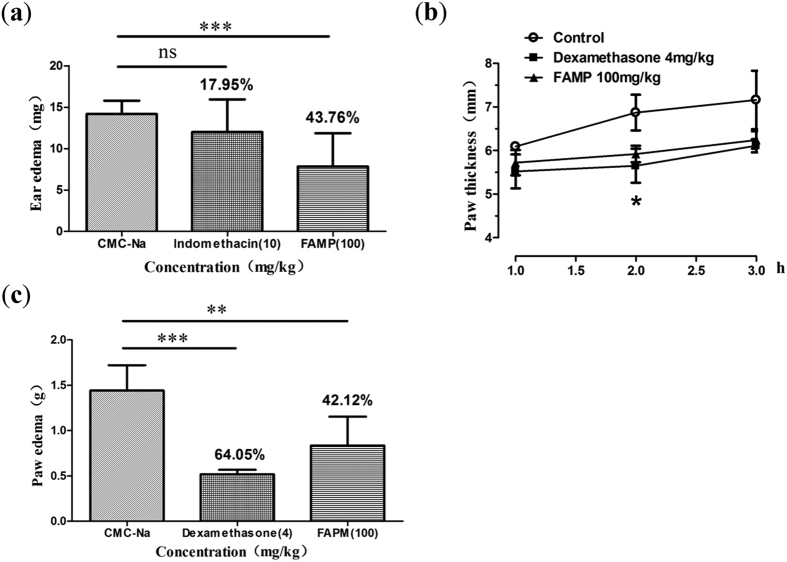
FAPM reduces inflammatory activities on mouse and rat swelling models. (**a**) Effects of FAPM on xylene-induced ear edema were shown. Animals were pretreated by FAMP (100 mg/kg), Indomethacin (10 mg/kg) or vehicle 60 min before the test (n = 10). (**b**) Effects-time curves of FAPM on Carr-induced paw edema. (**c**) Effects of FAPM on Carr-induced paw edema. Animals were pretreated by FAMP (100 mg/kg), Dexamethasone (4 mg/kg) or vehicle for 3 consecutive days (n = 6). The values represent the mean ± S.E.M.; *P < 0.05; **P < 0.01; ***P < 0.001 *vs.* control (vehicle) group. Inhibition percentage of the edema is expressed at the top in the bar.

Prostaglandin release is thought to be a pathogenic factor associated with PD. Therefore, the anti-inflammatory activities of FAPM were evaluated using the mouse ear swelling model. As shown in Fig. 7a, treatment of mice with FAPM (100 mg/kg) significantly inhibited the xylene-induced ear edema by 43.76% (*p* < 0.001). Treatment with indomethacin (10 mg/kg) also reduced the ear edema for 17.95% (*p* > 0.05).

As expected, the volume of the injected hind paw of rats was increased by subplantar injection of carrageenan (edema), which peaked 3 h post-injection. The time course for the development of paw-edema following administration of FAPM (100 mg/kg, i.p.) is shown in Fig. 7b. Dexamethasonea (4 mg/kg, i.g.), a steroid used to treat many inflammatory and autoimmune conditions, was used as a positive control. Two agents were able to significantly reduce the paw edema at 2 and 3 h post-injection. The inhibition of paw swelling by FAPM and dexamethasone was 42.12% (*p* < 0.01) and 64.05% (*p* < 0.001) respectively (Fig. 7c).

### Effect of FAPM on a mouse model of PD

**Figure 8:**
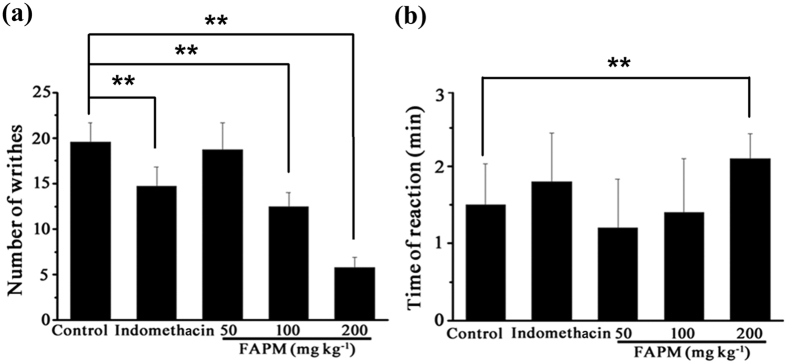
FAPM reduces PGF_2α_-induced pain response. (**a**) The number of writhes was counted for 30 min after PGF_2α_ administration. (**b**) The time to onset of writhing was recorded after PGF_2α_ administration. Results were expressed as means ± S.E.M (n = 10). **p < 0.01 *vs.* control (vehicle) group.

PGF_2α_ plays an important role in the pathogenesis of PD. PGF_2α_-induced writhing response occurred within 30 min after injection of PGF_2α_ in the females pretreated with estrogen while male mice did not experience any reactions. Administration of FAPM (100 and 200 mg/kg), 1 hour prior to PGF_2α_ injection, highlighted a significant and dose-dependent inhibition of PGF_2α_ -induced abdominal constrictions in mice (Fig. 8a). Indomethacin, the positive control, showed a significant inhibition of PGF_2α_-induced writhing response, similar to that of FAPM (100 mg/kg). The FAPM group (200 mg/kg) exhibited the maximum inhibiting effect of PGF_2α_-induced writhing (Fig. 8a) and a significantly increased latency time (*p* < 0.01) (Fig. 8b).

## Discussion

Protein tyrosine kinase/phosphatase activities contribute to the modulation of uterine contractions induced by contractile agonists or stretch via tyrosine phosphorylation and dephosphorylation reactions^12^,^13^,^14^,^15^. We previously reported that fluacrypyrim (FAPM) significantly increases the protein tyrosine phosphatases (PTPs) activity in a dose-dependent manner, inhibiting protein tyrosine phosphorylation which can be reversed by PTP inhibitor sodium pervanadate^20^. In the present study, we firstly demonstrated that FAPM treatment near completely inhibited PGF_2α_-, oxytocin-, Ach-, and high K^+^-induced uterine contractions in rats *in vitro*, with similar pD2 value from 5.72 to 5.92.

Uterine contraction is realized by the phosphorylation of 20 kDa myosin light chain (MLC_20_) at Ser^19^, stimulating the ATPase activity of the smooth muscle myosin^22^. The levels of MLC_20_ are regulated by opposing activities of MLC kinase (MLCK) and MLC phosphatase (MLCP)^23^. To stimulate MLCK by contractile agonists, such as PGF_2α_, oxytocin, *et al*., are mainly dependent upon increasing cytosolic Ca^2+^ and inhibiting MLCP^24^,^25^. As expected, we found FAPM, a novel inducer of PTPs, significantly reduced the PGF_2α_-induced MLC_20_ phosphorylation in dose-dependent manner, which indicated that FAPM inhibited smooth muscle contraction by affecting MLCK activity.

It has been reported that activated phospholipase C γ1 (PLC-γ1), produced in response to tyrosine phosphorylation, appears to be an important factor in uterine contractions, and Lck and c-Src kinases act an important role in regulating tyrosine phosphorylation of PLC-γ1 and uterine contractions in rats^14^,^15^. The sustained smooth muscle contraction induced by vascular smooth muscle cell membrane depolarization involves genistein-sensitive tyrosine phosphorylation, leading to phosphorylation of MYPT1 (the myosin-targeting subunit of MLCP) and MLC_20_^26^. The Ca^2+^-dependent tyrosine kinase Pyk2 (proline-rich tyrosine kinase 2) was then identified as the major tyrosine-phosphorylated protein in response to membrane depolarization^13^. Tyrosine kinase inhibitors, such as genistein, PP1, PP2, among others, attenuated uterine contractions elicited by the stimulation of contractile receptors, whereas the protein tyrosine phosphatase inhibitors, such as pervanadate, bpV(phen), *et al*., potentiated receptor-mediated contraction^15^,^16^. Hear we found FAPM, a novel inducer of PTPs, caused a concentration-dependent parallel rightward shift in the oxytocin-induced concentration–response curve without any reduction in maximal contractile response, demonstrating a competitive antagonism by FAPM against the oxytocin-induced contraction. These data indicated that inhibition of FAPM on myometrial MLC_20_ phosphorylation and contraction induced by contractile agents may be mediated through the increase of the PTPs activity.

Reversible oxidation of PTPs has emerged as an important regulatory mechanism whereby reactive oxygen species (ROS) inactivates PTPs, promotes phosphorylation and induction of the signaling cascade^27^,^28^. Mitochondrial reactive oxygen species (ROS), a major source of ROS in eukaryotic cells, are generally involved in oxidative stress, whereby complex III derived ROS especially has been involved in cellular redox signaling pathways^29^. However, a detailed understanding of the molecular mechanisms that trigger the generation of ‘signaling ROS’ from mitochondrial cytochrome bc1 complex is missing^30^. FAPM, with a β-methoxyacrylates structure, is proposed to be an inhibitor of mitochondrial cytochrome bc1 complex^31^. A structure–activity relationship assay indicated that the β-methoxyacrylates structure, a pharmacophore of class Ia inhibitors of mitochondrial cytochrome bc1 complex, is crucial for the inhibition of PGF_2α_-induced uterine contraction. At present, we do not know whether the effects of FAPM on uterine contraction are directly related to the inhibition of mitochondrial cytochrome bc1 complex. The biochemical mechanism by which FAPM operates is still needed and should be evaluated in future studies.

Clinical studies have demonstrated high PGF2a levels are associated with dysmenorrhea. Moreover, prostaglandin’s signaling has been shown to directly affect uterine contraction, and therefore may represent the most significant contributor to PD^6^,^32^. In China, the PGF_2α_ or oxytocin-induced writhing mouse models, represent frequently-used pain models of PD. We established this model and evaluated the anti-dysmenorrhea effects of FAPM. We noted that intraperitoneal administration of FAPM reduced the number of abdominal constrictions in a dose-dependent manner and increased the latency time when assessed in PGF_2α_-induced writhing in mice *in vivo*. These results indicate that FAPM may be potentially useful in the release of PD.

Inflammation is also believed to be involved in the pathogenesis of PD. A previous study reported that non-steroidal anti-inflammatory drugs could be utilized in the treatment of PD^8^. Several studies have evaluated the effect of a cyclooxygenase-2 (COX-2) inhibitor as a treatment for PD^33^,^34^. Our study evaluated the anti-inflammatory effects of FAPM, using classical xylene-induced ear-edema model^35^. As a chemical agent, xylene can cause severe vasodilation and edematous changes of skin partially associated with phospholipase A_2_ (PLA_2_)^35^,^36^. Our results indicate that FAPM significantly inhibited xylene-induced ear edema. This effect may be produced by inhibiting the release of PLA_2_ thereby reducing the concentrations of prostanoids and leukotrienes. For the further evaluation of the anti-inflammatory effects of FAPM, we performed a carrageenan-induced paw-edema assay in rats, which induces the development of edema in three phases: the early phase (the first 90 min) involving the release of histamine and serotonin; the second phase (90–150 min) mediated by kinin and the third phase (after 180 min) mediated by prostaglandin^35^,^37^. As shown in our results, FAPM significantly inhibited the development of the edemas induced by Carr after 2 h of treatment, while dexamethasonea, a steroidal anti-inflammatory agent as a positive control, inhibited the development of edema 1–3 h after treatment. Together these results further suggest that the anti-inflammatory mechanism of FAPM may be related to the inhibition of prostanoids and leukotrienes production.

The acetic acid-induced writhing test, an assay widely employed to assess anti-inflammatory and analgesic activities, is considered to be a model for visceral inflammatory pain^35^,^38^. It is generally thought that acetic acid lead to stimulation of nociceptive neurons by increasing endogenous mediators, such as prostaglandin and histamine, in peritoneal fluids. We found FAPM reduced the abdominal constrictions in a dose-dependent manner and increased the latency time when assessed in acetic acid-induced writhing in mice. These results suggest the effect of FAPM could be attributed, at least in part, to a reduction of inflammatory mediators.

Above all, we found FAPM, a novel inducer of PTPs, significantly reduced the PGF_2α_-induced MLC_20_ phosphorylation, near completely inhibited PGF_2α_-, oxytocin-, Ach-, and high K^+^-induced uterine contractions in rats *in vitro*, and caused a concentration-dependent parallel rightward shift in the oxytocin-induced concentration–response curve. In consistent with the *in vitro* activity, we demonstrated FAPM dose-dependently reduced the number of abdominal constrictions and increased the latency time in PGF_2α_- and acetic acid-induced writhing test in mice *in vivo*. It was then found that FAPM inhibited the development of Carr-induced rat paw edemas and thexylene-induced mouse ear edemas. A structure–activity relationship assay based on the inhibition of PGF_2α_-induced uterine contraction indicated that the mitochondrial cytochrome bc1 complex may be the target of FAPM for its uterine relaxant effect as well as its antinociceptive and anti-inflammatory activity, through the inhibition of ROS production from mitochondria. Therefore, rational modifications of FAPM by medicinal chemistry techniques are anticipated to obtain derivative uterine relaxants with better specificity and therapeutic potential for patients suffering from PD.

## Materials and Methods

### Materials

PGF_2α_, Oxytocin, acetylcholine (Ach), indomethacin, Xylene, Carrageenan and dexamethasone were purchased from Sigma (St. Louis. MO). Actin antibody, phospho-myosin light chain-20 (p-MLC_20_, ser19) antibody and HRP goat anti-rabbit IgG were obtained from Cell Signaling Technology (Beverly, MA). Fluacrypyrim (FAPM) and its analogs were synthesized by Dr. Yang’s lab. Stock solutions were prepared in DMSO. Estrostilben was obtained from Beijing Yimen Co. Ltd and dissolved in 0.9% sodium chloride.

### Ethics StatementN

This study was approved by Beijing Experimental Animal Ethics Committee (2006) No. 5118 set by the Beijing People’s Government.

### Animals

SPF KM male and female mice, weighting from 22 to 28 g, and female Sprague Dawley (SD) rats, weighing 120–140 g were purchased from the Laboratory Animal Center, Chinese Academy of Medical Sciences. The animals were kept in an environmentally controlled room at 22 ± 2 °C with free access to pellet food and water on a 12-h light/dark cycle. All animal procedures were performed in accordance with the National Institutes of Health guidelines for the care and use of laboratory animals. The method of euthanasia was cervical dislocation and the method of anesthesia which we used in some tests was intraperitoneal administration of sodium pentobarbital.

### Assessment of Uterine Contractility *in vitro*

Female SD rats were administered intraperitoneally with estradiol benzoate (0.1 mg/kg) for 2 days before the experiments. Rats were sacrificed by cervical dislocation. Uteri were removed and then cut into 10 × 2 × 2 mm^3^ strips along the longitudinal axis. The uterine strips mounted vertically in an organ bath containing 5 ml organ baths of Krebs’ solution (136 mM NaCl, 2.68 mM KCl, 1.8 mM CaCl_2_, 0.5 mM MgCl_2_, 11.9 mM NaHCO_3_, 0.32 mM NaH_2_PO_4_ and 5.04 mM glucose, pH 7.2), aerated continuously with 95% O_2_/5% CO_2_ and maintained at 37 ± 0.2 °C. The tension of the myometrial rings was measured isometrically with a tension transducer connected to a polygraph system (Beijing Microsignalstar Techology Development Co., Ltd, China). To ensure contractile viability and to determine maximum contraction, the solution for each strip was first changed to 40 mM K^+^ for 10 min. The strips which did not respond to KC1 were discarded. The recorded value to 40 mM K^+^ for 10 min was taken as the control^39^. Each uterine strip was allowed to equilibrate at 1 g tension for 20 min until a steady tension was achieved and then treated with different agents (PGF_2α_, oxytocin, Ach, or KCl) to stimulate uterine contraction. Various concentrations of FAPM were then added to the bath solution in a cumulative manner at 10-min intervals. Mechanical responses of uterine strips were analyzed by the area under the curve (AUC) according to the response curve of each uterine strip tested and expressed as a percentage of the control.

### Antagonism assasyment of oxytocin-induced contraction

As described above, uterine strips were prepared and disposed before the experiment. Each uterine strip was allowed to equilibrate at 1 g tension for 20 min until a steady state and then treated with vehicle and FAPM of 0.25 μM, 0.5 μM and 1 μM. Various concentrations of oxytocin were then added to the bath solution in a cumulative manner at 10-min intervals. Mechanical responses of uterine strips were analyzed by the area under the curve (AUC) according to the response curve of each uterine strip tested and expressed as a percentage of the control. EC_50_ of oxytocin for each group was calculated and the Schild regression analysis was performed using the following formula:


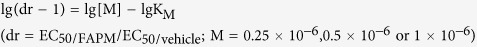


And pA_2_ = lgK_M_ when lg(dr − 1) = 0 in the linear plot.

### Culture of Dispersed Myometrial Cells

Primary cultures of uterine smooth muscle cells were prepared as previously described by us^25^. Briefly, uteri was removed from estrostilben primed rats and dissected free of fat and endometrium. The tissues were cut into 1 × 1 × 1 mm^3^ pieces and placed in culture flasks containing DMEM medium supplemented with 15% fetal bovine serum (FBS) and maintained at 37 °C in 5% CO_2_ atmosphere. Cells were subcultured every 3–4 days prior to reaching confluence. When most of the cells had contracted and become rounded, the semi-dispersed cells were washed with PBS twice, and trypsin (0.25% w/v) was added. FBS was added to neutralize the effect. The dissociated myometrial cells were collected by centrifugation (200 *g* for 15 min) and resuspended in DMEM medium containing 10% FBS, then plated on glass coverslips for 24 h at 37 °C in 5% CO_2_ atmosphere before the experiments.

### Total Protein Extraction

Protein extraction was performed according to the method as previously described by us^25^. Briefly, myometrial cells were plated at a cell density of 5 × 10^4^ cells/well in 60-mm culture plates and cultured in serum-free DMEM for 24 h. After treatment with varying concentrations of FAPM and PGF_2α_ for 5 min, myometrial cells were washed with cold PBS three times, and then 2 × SDS loading buffer was added. The cell lysates were then boiled for 10 min and pelleted by centrifugation (10,000 g, 5 min). The samples were aliquoted and stored at −20 °C for further use.

### MLC_20_ Phosphorylation Analysis

MLC_20_ phosphorylation was analyzed by Western blot as previously described^25^. In brief, the cell lysates were centrifugated at 12,000 × *g* for 10 min at 4 °C. Equal amounts of protein (20 μg) were separated on 12% SDS-polyacrylamide gel electrophoresis (SDS-PAGE) and then transferred to nitrocellulose membranes. The membranes were probed with the phospho-Ser-19-specific antibody, followed by an HRP-conjugated secondary antibody. The enhanced chemiluminescence system detection kit (Cell Signaling) was used to visualize the immunoreactive proteins on PVDF membranes.

### Acetic acid-induced abdominal writhing in mice

Acetic acid-induced writhing test was performed according to the previously described methods with minor modification^35^. Fifty mice were divided randomly into five groups. One hour before the test, a single dose of FAPM (50, 100 and 200 mg/kg) and indomethacin (50 mg/kg) were administered intraperitoneally. The mice were then injected intraperitoneally with acetic acid (0.6%, v/v in saline, 10 ml/kg b w., i.p.) and placed in separated boxes. The number of abdominal writhing was recorded for 30 min and the time that passed until the first appearance of writhing was recorded as the latency of pain response. For the precision and accuracy of the results, two well-trained lab technicians performed the test in a double-blind manner.

### Xylene-induced ear edema in mice

The xylene-induced ear edema test was performed according to the previously described methods^38^. In brief, fifty mice were divided randomly into five groups. 1 h before the test, a single dose of FAPM (50, 100 and 200 mg/kg) and indomethacin (50 mg/kg) were administered intraperitoneally. The test animal was received 20 μL xylene to the inner and outer surface of the right ear, while the left ear kept untreated. One hour later, mice were sacrificed and the central sections of the right and left ears were obtained using a stainless steel 6 mm punch. The weight difference between the sections of the right and left ears was used to evaluate ear edema. The ear edema percent was calculated using the following formula:


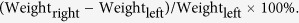


### Carrageenan induced paw edema in rats

Carrageenan induced paw edema in rats was conducted according to the method described previously^35^, with minor modification. Eighty rats were divided randomly into three groups. Before edema induction, the test groups were orally administered with dexamethasone (4 mg/kg), intraperitoneally injected with FAPM (100 mg/kg) and vehicle (DMSO, 20%) for 3 consecutive days. 30 min after the last administration, the rat left hind paw was injected sub-plantar with 0.1 ml of 1% carrageenan to induce edema, while the right hind paw kept untreated. Paw thickness was measured with a digital micrometer at 0, 1, 2 and 3 hours following injection. The rats were sacrificed at 3 h after carrageenan injection. Both hind paws were removed from the knee joint and weighed with an analytical balance. An increase in paw weight (in g) between the treated and the untreated paw was used to evaluate the degree of the hind paw swelling and the inhibition percent of paw swelling was calculated by using the following equation:


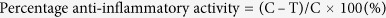


where C and T indicate the non-treated and drug treated edema, respectively.

### *In vivo* mouse model of PD

In brief, fifty female mice were randomly separated into five groups. Before the test, each mouse was intragastrically treated with 0.2 mg diethylstilbestrol daily for 12 days. On the twelfth day, 30 min after the last administration, the test groups were intraperitoneally injected with FAPM (50, 100 and 200 mg/kg), indomethacin (50 mg/kg) and vehicle (DMSO, 20%). 1 hour after drug administration, the mice were treated with PGF2α (i.p. 1.3 mg/kg) and placed in separated boxes. The writhing responses were observed for 30 min and the time that passed until the first appearance of writhing was recorded as the latency of pain response. For the precision and accuracy of the results, two well-trained lab technicians performed the test in a double-blind manner.

### Statistical Analysis

Results are expressed as means + /−SEM for n samples, and statistical analyses were performed using the GraphPad Prism 5 software (GraphPad Software, San Diego, CA). Student’s t-test was used for comparison of the means of two groups. One-factor ANOVA followed by Dunnett-t test was applied for MLC_20_ phosphorylation analysis (Fig. 5b), acetic acid-induced abdominal writhing assay (Fig. 6) and PGF_2α_-induced pain response assay (Fig. 8). Two-factor ANOVA followed by Student-New-Keuls (SNK-q) test was applied for the inhibition experiment of uterine contraction (Figs 1c, 2b and 4b,c) and paw edema assay (Fig. 7b). Values of P < 0.05 were considered significant.

## Additional Information

**How to cite this article:** Li, Z. *et al*. Flucrypyrim, a novel uterine relaxant, has antinociceptive and anti-inflammatory effects *in vivo. Sci. Rep.* **7**, 42040; doi: 10.1038/srep42040 (2017).

**Publisher's note:** Springer Nature remains neutral with regard to jurisdictional claims in published maps and institutional affiliations.
